# Classes of childhood adversities and their associations to the mental health of college undergraduates: a nationwide cross-sectional study

**DOI:** 10.1186/s12199-021-00993-7

**Published:** 2021-07-17

**Authors:** Peigang Wang, Mohammedhamid Osman Kelifa, Bin Yu, Yinmei Yang

**Affiliations:** 1 School of Health Sciences, Wuhan University, 115 Donghu Road, Wuhan City, 430071 Hubei Province China; 2 Orotta College of Medicine and Health Sciences, Asmara, Meakel Eritrea; 3 Department of Surgery, Duke University, Durham, NC 27710 USA

**Keywords:** Adverse childhood experiences, Current stressful events, Psychological distress, Subjective well-being

## Abstract

**Background:**

Childhood adversities pose deleterious consequences on health and well-being, but limited studies explore whether unique patterns of adverse childhood experiences (ACEs) impact the mental health of emerging adults and the mediating role of current stressful events (CSEs). This study examined classes of ACEs and how they relate to CSEs, psychological distress, and subjective well-being among Eritrean College undergraduates.

**Methods:**

Cross-sectional data on ACEs, CSEs, symptoms of psychological distress, and subjective well-being were collected from a national sample of college students (*N* = 507). We identified ACE patterns using latent class analysis and further examined whether CSEs mediated the effects of ACE classes on psychological distress and subjective well-being.

**Results:**

86.4% of the sample experienced at least one ACE. Collective violence, domestic violence, and physical abuse were the most common ACEs. Three subgroups, low ACEs (66.3%), household violence (19.1%), and multiple ACEs (14.6%) were identified. We found that relative to low ACEs, household violence (β = 0.142, 95% CI 0.046, 0.248) and multiple ACEs (β = 0.501, 95% CI 0.357, 0.666) indirectly influenced psychological distress through CSEs, and CSEs mediated the relationships between household violence (β = −0.096, 95% CI −0.176, −0.033), multiple ACEs (β = −0.338, 95% CI −0.498, −0.210), and subjective well-being. However, there were nonsignificant relative direct effects of ACE patterns on both psychological distress and subjective well-being.

**Conclusions:**

Experiencing multiple ACEs and household violence in conjunction with CSEs significantly predict psychological distress and subjective well-being. Contextual interventions for the early identification of ACEs and the management of CSEs may play a crucial role in the prevention of mental health problems.

**Supplementary Information:**

The online version contains supplementary material available at 10.1186/s12199-021-00993-7.

## Background

The mental health of undergraduate college students has gained a public health interest, as the demand for mental health services has increased, especially in settings with limited resources such as Eritrea. Psychological distress including anxiety, depression, suicidality, and somatic symptoms is a common health problem among college students [1, 2], and compared to the general population, college students regularly report higher levels of psychological distress [3]. Likewise, subjective well-being tends to decline among students after joining college [4].

The World Mental Health Surveys International College Student Project, for instance, reported that 38.4% of college students screened positive for at least one mental disorder [5]. Moreover, about 43% of students with at least one mental illness reported difficulty functioning [6], disrupting their academic performance, well-being, social life, and employment [7].

In terms of social and economic values, undergraduate college students represent an important segment of emerging adults (18–25 years), and they undergo distinct psychosocial developments [8]. The proportion of emerging adults is more than 30% of the Eritrean population [9]. Self-focus, instability (in-between self-reliance and dependence), and exploration of identity together with the feelings of great hopes and uncertainties are characteristic of this age group [10]. These developmental processes coupled with academic pressures and the requisite to adjust to new social and physical conditions might entail sources of stress for students [11]. These current stressful events (CSEs) occur within a limited period (few weeks to a year), including work strains and safety, financial problems, interpersonal relationships, family and personal conflicts, educational issues, and health-related stressors [12, 13]. CSEs are linked to poor subjective well-being and psychological distress [14, 15]. Generally, CSEs are the closest antecedents of psychological distress. For instance, depressed people were 2 to 9 times more likely to report a stressful life event before the onset of a depressive episode [16]. Stress during the transition to college also negatively affects the successful transition and subjective well-being of college students, particularly among students with a history of adverse childhood experiences (ACEs). ACEs are well-known remote risk factors for adult mental health problems [17], which refers to “childhood events, varying in severity and often chronic, occurring in a child’s family or social environment that cause harm or distress, thereby disturbing the child’s physical or psychological health and development” [18]. Researchers documented that 39% of college students reported at least one ACE [19], and students with histories of ACEs were more likely to report symptoms of psychological distress than those with no histories [20, 21].

The pathways linking ACEs to future stress and negative outcomes are complex, despite the far-reaching impact of ACEs on adult psychological distress is supported by substantial evidence from both prospective and retrospective research [22, 23]. Additionally, a 2-year follow-up population study revealed that ACEs predisposed women to depression, but CSEs increased the likelihood of depression on follow-up [24]. In a sample of undergraduates, both ACEs and CSEs significantly predicted mental health, with CSEs exerting a greater contribution [21]. Further, a cross-sectional study among a sample of young adults found that ACEs and adult stress significantly predicted psychological distress, with adult stress as a mediator [25].

The stress sensitization and stress proliferation frameworks may provide theoretical foundations for the current study. The stress sensitization hypothesis asserts that ACEs disrupt the normal development of brain areas responsible for stress regulations [26] and lower the threshold for future stressors [27]. Toxic stress from early life not only leads to mental health problems but also triggers chains of other stressors that compromise health and recovery during adulthood. According to stress proliferation theory, early adverse experiences lead to subsequent new stressors that were not previously present [28]. The stress sensitization and stress proliferation models undertake a life-course perspective to better appreciate the long-term impact of early-life adversity on adulthood stressful life events and subsequent mental health.

Research regarding ACEs typically employed the cumulative risk approach, and it assumes that ACE types have equal weight and an additive and linear dose-response relationship to health outcomes. However, evidence shows that some ACE types (e.g., sexual abuse) exert stronger impacts on outcomes than others [29–31], and ACEs tend to occur in multiple rather than single experiences [32]. Hence, this method precludes the identification of potential heterogeneity of ACE categories.

The person-centered approach or latent class analysis (LCA) has an advantage over the cumulative-risk approach in discerning potential subgroups of ACEs, which in turn, may exert different effects on health outcomes [33–36]. Some studies using LCA have discovered distinctive patterns of ACEs. Most of these studies identified three or four classes commonly containing one class with low ACEs, another one with a high incidence of ACEs, and latent classes differentially predicted outcomes. The types of ACEs that constitute each class extensively vary across studies, which could be attributed to different environmental and sociocultural contexts [37]. For example, a study indicated that household violence class characterized by higher rates of physical abuse, emotional abuse, and witnessing domestic violence was common among university students in four East Asian regions (Japan, China, Taiwan, and Hong Kong) [38]. More so, while the ACEs class comprising parental mental illness and divorce, substance/alcohol abuse, and poly-victimization was common among Dutch and US children [39, 40], a cluster of community violence and economic disadvantages was peculiar to Burkina Faso children [41], indicating variation in types of risks across contexts.

Contexts (e.g., cultural, economic, and political) do not only affect the occurrence of ACEs but also how individuals interpret and cope with adversities [42]. Corporal punishment, for instance, may have different meanings in Sub-Sahara African families than that of western families [43]. For example, Eritrean parents are more authoritarian towards their children and deeply interfere in all aspects of their lives even when they reach adulthood [44]. Likewise, children grow up in wide social networks of extended families where religious and cultural beliefs play significant roles in the daily lives and well-being of people [45]. On the other hand, since the end of the World War II, Eritrea has experienced a brief period of peace and stability (1991–1997). The Ethiopian war of 1998–2000 marked the start of Eritrea’s no-war, no-peace situation, which has culminated in a poor socioeconomic situation. Our participants were either born during the active war or grew up afterward, making them a high-risk population for ACEs and psychological trauma due to the persistent stressors of war and its aftermath. Although ACEs are deemed prevalent in similar settings, little is known about ACE patterns and how they relate to CSEs, psychological distress, and subjective well-being among emerging adults in this setting, particularly relevant ACE types such as community and collective violence are rarely investigated in most ACE studies. Furthermore, the relationship between ACEs and CSEs is an ongoing area of research activity, which could be more relevant to settings affected by war. Hence, on top of addressing this literature gap, identifying individuals who are vulnerable to specific classes of ACEs and how these patterns relate to adulthood stressors and mental health outcomes is crucial to prioritize contextually relevant prevention and intervention strategies.

Accordingly, the current study identified latent classes of ACEs and examined their association to subjective well-being and psychological distress among Eritrean undergraduate students. We assumed that undergraduate college students in Eritrea were exposed to high rates of childhood adversities than proportions established in other samples. Additionally, our second hypothesis was that LCA would identify different ACE patterns. Finally, we hypothesized that CSEs would mediate the effects of ACE patterns on psychological distress and subjective well-being.

## Methods

### Procedures and participants

5740 undergraduate students were registered in seven colleges of the Eritrean institute of higher education and research in 2018. To draw a representative sample, first, we stratified students in all colleges by their respective year of study (years 1–6). Next, participants were selected through a systematic sampling from an alphabetically arranged name list, with a sample interval of 10. Cross-sectional data were obtained using self-administered and pencil-paper-based questionnaires. Questionnaires were written in English (medium of instruction in Eritrea), and unfamiliar terminology was also translated from English into Tigrigna (a widely spoken language in Eritrea). Respective Institutional Review Boards approved the study, and all participants provided written informed consent. A total of 564 students participated in this survey. We excluded participants who failed to respond to the key variables (n = 45). As the current study mainly focuses on emerging adults (18–25 years), we further excluded participants aged less than 18 or over 25 (n = 12). Finally, 507 respondents were eligible for analysis, indicating a response rate of 89.9%.

### Measures

#### Adverse childhood experiences

The Adverse Childhood Experience International Questionnaire (ACE-IQ), the frequency version, was utilized to capture self-reported childhood adversities [46]. It has been a reliable measure of ACEs in a similar setting (South Africa) [47]. This 29-item instrument assesses exposure to 13 categories of ACEs including physical abuse (A3-A4), emotional abuse (A1-A2), sexual abuse (A5-A8), household substance abuse (F1), incarcerated family member (F3), household mental illness (F2), domestic violence (F6-F8), parental separation/divorce (F4-F5), emotional neglect (P1-P2), physical neglect (P3-P5), bullying (V1), community violence (V4-V6), and collective violence (V7-V10) (see Appendix 1). We further dichotomized ACE categories, which differs by ACE types (e.g., exposure to contact sexual abuse only requires being touched in a sexual way once, whereas exposure to bullying requires being bullied many times). The current study modified the scoring of physical neglect and physical abuse, as only 5.5% of college students reported physical neglect or physical abuse based on the user guide. However, child physical abuse and neglect are prevalent and widely complicated by structural and deeply rooted sociocultural norms in Eritrea [48]. The scoring of physical neglect and physical abuse were modified to reflect the real situations in Eritrea. Therefore, we recoded responses of “A Few Times” or “Many Times” as exposure to physical neglect and physical abuse (coded as 1). Finally, the total number of ACE categories was summed to form a cumulative score, ranging from 0 to 13. Tetrachoric correlations between ACE categories ranged from −0.01 to 0.64 (see Appendix 2).

#### Current stressful events

Current stressful events in the last 12 months were measured using the college student’s Stressful Event Checklist [49, 50]. Items were rated on 20 dichotomous variables (yes/no), such as “having ongoing conflicts with parents,” “unwanted pregnancy (either you or as the father),” and academic and general life circumstances (e.g., illness, injury, drug abuse) (see Appendix 3). We summed the number of “Yes” responses to yield a scale score. Tetrachoric correlations between CSE items ranged from −0.12 to 0.82 (see Appendix 4).

#### Psychological distress

Symptomology of psychological distress was assessed using the Self-Reporting Questionnaire (SRQ-20) (yes/no) [51], a widely employed instrument to screen for symptoms of psychological distress, including depression, anxiety-related disorders, and somatoform disorders. It has been validated in the Eritrean adult population (α = 0.78) [52]. Participants reported whether they experienced certain feelings in the last 30 days, e.g., “Do you often have headaches?” “Do you feel unhappy?” “Yes” responses were summed to yield a composite score (range 0–20), with higher scores reflecting more symptoms of psychological distress. Tetrachoric correlations between SRQ-20 items ranged from 0.06 to 0.64 (see Appendix 5).

#### Subjective well-being

Subjective well-being was self-reported using the 5-item World Health Organization Well-Being Index (WHO-5) [53]. This scale reflects basic subjective states of well-being, such as positive mood, vitality, and general interests [54]. Participants rated these items (e.g., “I woke up feeling fresh and rested in the last two weeks”) on a 6-point Likert scale ranging from 0 (at no time) to 5 (all of the time). Higher scores represented better subjective well-being. It has been widely used worldwide [55], and this scale demonstrated a Cronbach’s alpha of 0.82 in the current study.

### Statistical analysis

First, we evaluated tetrachoric correlations of ACE categories, CSE items, and psychological distress items. To determine the correlations between the key study variables, we also examined Pearson correlations between continuous variables. Then, models with one through five latent classes were conducted using the 13 ACE categories. The optimal model was chosen on a combination of Akaike Information Criteria (AIC), Bayesian Information Criteria (BIC), Adjusted Bayesian Information Criteria (aBIC), Entropy, the Bootstrap Likelihood Ratio Test (BLRT), class size, and interpretability [56]. Moreover, after identifying the optimal number of classes, students were assigned to ACE latent classes using the most likely class membership. Following Hayes and Preacher [57], we standardized all continuous variables (CSEs, psychological distress, and subjective well-being) before path analysis. Then, we examined the potential mediating role of CSEs in the relationships between ACEs latent classes (multicategorical independent variable) and the distal outcomes (psychological distress and subjective well-being). To obtain estimates of 95% bias-corrected bootstrap confidence interval (CI), we used the bootstrap method (based on 10,000 bootstrap samples). Tetrachoric correlations, LCA, and mediation tests were conducted using Mplus 8.0, whereas all other analyses were performed with SPSS 23.0. *p* < 0.05 (two-tailed) was statistically significant.

## Results

### Descriptive statistics

Participants in this study comprised of 50.5% males, and the mean age was 19.7 years (SD = 1.5). Means and standard deviations of ACEs, CSEs, psychological distress, and subjective well-being are listed in Table 1. 86.4% (438/507) of the sample experienced at least one ACE. Collective violence (40.2%), domestic violence (38.7%), and physical abuse (36.7%) were the most common ACEs, while household substance abuse (4.9%) was the least prevalent. The Pearson correlations for ACEs, CSEs, subjective well-being, and psychological distress were significant at *p* < 0.001 level (see Table 2). Specifically, ACEs were negatively related to subjective well-being (*r* = −0.16) and positively correlated to psychological distress (*r* = 0.26) and CSEs (*r* = 0.47). CSEs were negatively related to subjective well-being (r = −0.30), whereas CSEs were positively correlated to psychological distress (*r* = 0.45).

**Table 1:** Descriptive statistics (*N* = 507)

Variables	Mean (SD)	Range
Age	19.7 (1.5)	18–25
ACEs	2.7 (2.2)	0–12
CSEs	4.5 (2.7)	0–14
Psychological distress	6.2 (4.3)	0–20
Subjective well-being	14.8 (5.2)	0–25
Category	N	%
Gender (male)	256	50.5
Physical abuse	186	36.7
Emotional abuse	45	8.9
Sexual abuse	137	27.0
Household substance abuse	25	4.9
Incarcerated family member	60	11.8
Household mental illness	32	6.3
Domestic violence	196	38.7
Parental separation/divorce	115	22.7
Emotional neglect	97	19.1
Physical neglect	89	17.6
Bullying	32	6.3
Community violence	157	31.0
Collective violence	204	40.2

*ACEs* adverse childhood experiences, *CSEs* current stressful events, *SD* standard deviation

**Table 2:** Pearson correlations between the study variables

	ACEs	Subjective well-being	Psychological distress	CSEs
ACEs	1			
Subjective well-being	−0.16^***^	1		
Psychological distress	0.26^***^	−0.51^***^	1	
CSEs	0.47^***^	−0.30^***^	0.45^***^	1

^***^p < 0.001

*ACEs* adverse childhood experiences, *CSEs* current stressful events

### Number of latent classes

Model selection statistics with one through five latent classes is summarized in Table 3. The AIC decreased as the number of classes increased, and the two-class model had the lowest BIC and aBIC. Overall, the three-class model had the highest Entropy, relatively small AIC, BIC, and aBIC. Additionally, after checking the item-response probabilities, we found that the three-class model created three meaningful and distinct classes, but one class in the four-class model was not interpretable. Furthermore, BLRT in the four-class model with a nonsignificant *p* value indicated the three-class model was preferable. The smallest class consisted of 14.6% of the sample for the three-class model. Consequently, the 3-solution model was selected on a combination of model fit and interpretability.

**Table 3:** Model selection statistics

#Classes	AIC	BIC	aBIC	Entropy	*p*-BLRT	Class size (%)
1	6129.770	6184.740	6143.477	-	-	-
2	5841.776	5955.945	5870.244	0.740	< 0.001	75.1/24.9
**3**	**5830.329**	**6003.698**	**5873.560**	**0.768**	**< 0.001**	**14.6/66.3/19.1**
4	5820.996	6053.564	5878.987	0.701	0.154	52.7/23.5/8.9/15.0
5	5818.819	6110.586	5891.572	0.726	0.375	9.5/3.5/26.8/10.8/49.5

Accepted model is presented in boldface

*AIC* Akaike’s Information Criteria, *BIC* Bayesian Information Criteria, *aBIC* Adjusted Bayesian Information Criteria, *BLRT* Bootstrap Likelihood Ratio Test

### Item-response probabilities and latent class prevalence

**Fig. 1:**
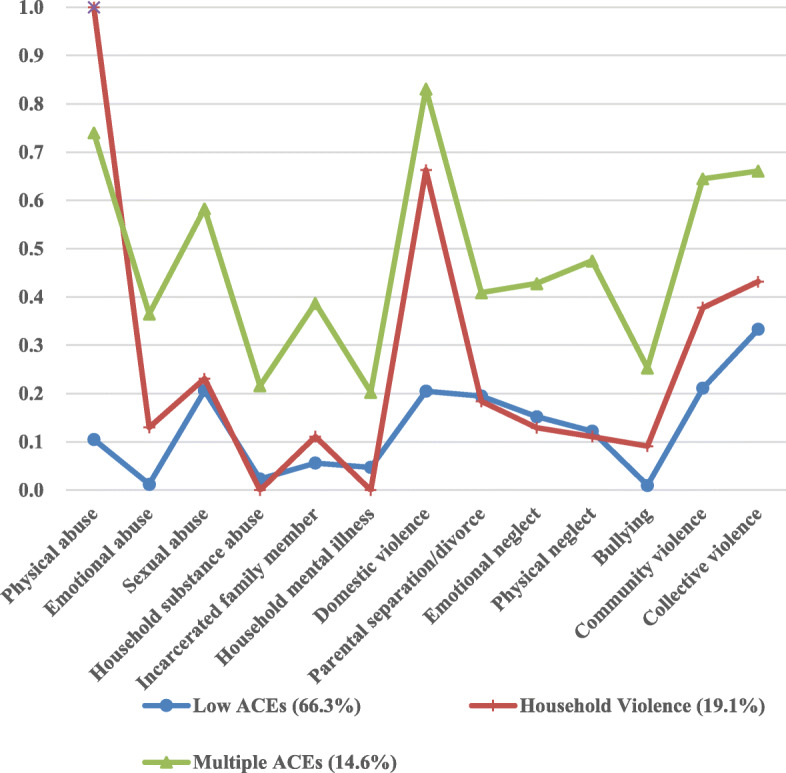
Patterns of ACEs among college undergraduates. *ACEs* adverse childhood experiences

As shown in Fig. 1, three distinctive classes were identified: low ACEs (66.3%), household violence (19.1%), and multiple ACEs (14.6%). Those in the low ACE class were characterized by relatively low probabilities of ACE exposures. In contrast, individuals in the multiple ACEs class were marked by high odds of all ACE categories. Participants in the household violence class reported considerably high likelihoods of endorsing exposures to physical abuse (100.0%) and domestic violence (66.3%) but were less likely to report other categories. Individuals in the multiple ACEs class had the cumulative ACE score at 6.6 (SD = 1.4), higher than those in the household violence (M = 3.5, SD = 1.0) and low ACEs (M = 1.6, SD = 1.2) classes.

### The mediation effect of CSEs on the relationship between ACEs latent classes and mental health

**Fig. 2:**
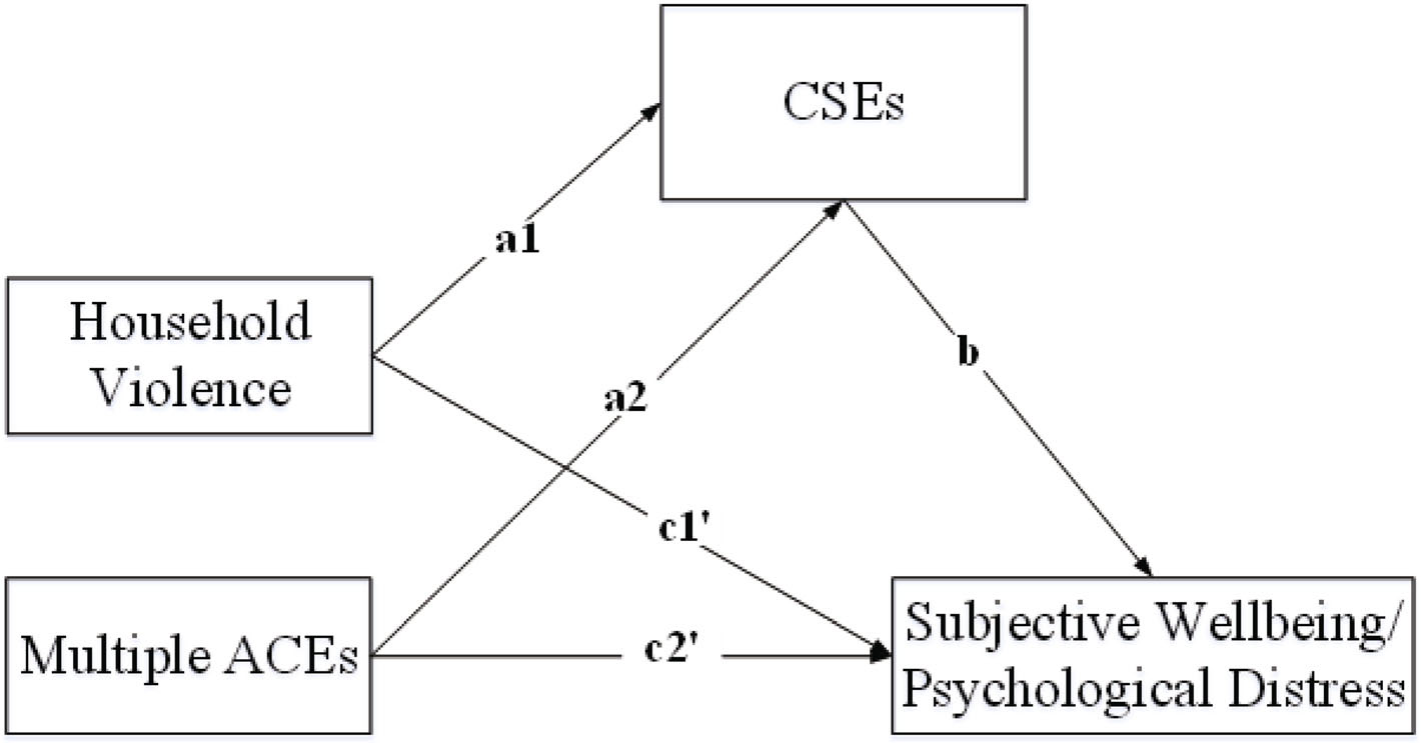
Multicategorical mediation model for psychological distress/subjective well-being. a1 and a2 represent the effects of household violence and multiple ACEs on CSEs, compared to low ACEs; b represents the effect of CSEs on psychological distress/subjective well-being; c1' and c2' represent the relative direct effects of household violence and multiple ACEs on psychological distress/subjective well-being, relative to low ACEs. *CSEs* current stressful events, *ACEs* adverse childhood experiences. All paths are unstandardized coefficients

Dummy codes were created for household violence and multiple ACEs with low ACEs as the reference group. As depicted in Fig. 2, a multicategorical mediation model was examined.

As shown in Tables 4 and 5, relative to low ACEs, students endorsing household violence (β = 0.317, *p* = 0.003) and multiple ACEs (β = 1.117, *p* < 0.001) reported higher levels of CSEs, which in turn increased psychological distress (β = 0.448, *p* < 0.001) and decreased subjective well-being (β = −0.302, *p* < 0.001). The relative direct effects of household violence and multiple ACEs on psychological distress and subjective well-being were nonsignificant. We also found that relative to low ACEs, CSEs significantly mediated the effects of household violence and multiple ACEs on psychological distress and subjective well-being, and the mediation effects of CSEs on psychological distress and subjective well-being were stronger in the multiple ACE class than in the household violence class.

**Table 4:** Multicategorical mediation model: the mediating role of CSEs on the relationship between household violence, multiple ACEs, and psychological distress, relative to low ACEs (*N* = 507)

Pathways	Unstandardized β (SE)	*p* value	Bias corrected 95% CI
CSEs on			Lower, upper
Household violence (a1)	0.317 (0.108)	0.003	0.105, 0.528
Multiple ACEs (a2)	1.117 (0.138)	<0.001	0.849, 1.395
Psychological distress on			
CSEs (b)	0.448 (0.046)	<0.001	0.359, 0.538
Household violence (c1’)	−0.096 (0.099)	0.333	−0.295, 0.092
Multiple ACEs (c2’)	0.014 (0.129)	0.916	−0.232, 0.275
Relative indirect effects			
a1*b	0.142 (0.051)	0.005	0.046, 0.248
a2*b	0.501 (0.079)	<0.001	0.357, 0.666
Relative total effects			
a1*b+ c1'	0.046 (0.109)	0.674	−0.171, 0.256
a2*b+ c2'	0.515 (0.128)	<0.001	0.265, 0.773

a1 and a2 represent the effects of household violence and multiple ACEs on CSEs, compared to low ACEs; b represents the effect of CSEs on psychological distress; c1' and c2' represent the relative direct effects of household violence and multiple ACEs on psychological distress, relative to low ACEs; a1*b and a2*b represent the relative indirect effect on psychological distress through CSEs for household violence and multiple ACEs, compared to low ACEs

*CSEs* current stressful events, *ACEs* adverse childhood experiences, *CI* confidence interval, *SE* standard error

**Table 5:** Multicategorical mediation model: the mediating role of CSEs on the relationship between household violence, multiple ACEs, and subjective well-being, relative to low ACEs (*N* = 507)

Pathways	Unstandardized β (SE)	*p* value	Bias corrected 95% CI
CSEs on			Lower, upper
Household violence (a1)	0.317 (0.108)	0.003	0.105, 0.528
Multiple ACEs (a2)	1.117 (0.138)	<0.001	0.849, 1.395
Subjective well-being on			
CSEs (b)	−0.302 (0.049)	<0.001	−0.397, −0.202
Household violence (c1')	0.073 (0.114)	0.526	−0.154, 0.294
Multiple ACEs (c2')	0.030 (0.145)	0.839	−0.263, 0.310
Relative indirect effects			
a1*b	−0.096 (0.036)	0.008	−0.176, −0.033
a2*b	−0.338 (0.073)	<0.001	−0.498, −0.210
Relative total effects			
a1*b+ c1'	−0.023 (0.119)	0.844	−0.261, 0.203
a2*b+ c2'	−0.308 (0.133)	0.021	−0.571, −0.049

a1 and a2 represent the effects of household violence and multiple ACEs on CSEs, compared to low ACEs; b represents the effect of CSEs on subjective well-being; c1' and c2' represent the relative direct effects of household violence and multiple ACEs on subjective well-being, relative to low ACEs; a1*b and a2*b represent the relative indirect effect on subjective well-being through CSEs for household violence and multiple ACEs, compared to low ACEs

*CSEs* current stressful events, *ACEs* adverse childhood experiences, *CI* confidence interval, *SE* standard error

## Discussion

86.4% of study participants reported at least one ACEs, higher than previous rates among college students in East Asia (66.27%), Tunisia (74.8%), and Vietnam (76%) [38, 58, 59]. High exposures to ACEs of young people in Eritrea might have arisen from a combination of multiple wars, political instability, and their socioeconomic associates [60].

We identified three distinct subgroups, including low ACEs, multiple ACEs, and household violence. The multiple ACEs class was characterized by the highest cumulative score of ACEs, consistent with prior studies [30, 61–63]. Findings from previous studies typically reported three or four latent classes, commonly one class with relatively low probabilities of ACE exposures, and another with high odds of ACE categories [35, 64, 65]. Additionally, compared to previous findings [66, 67], we identified a novel class (household violence class) characterized by frequent occurrences of physical abuse and domestic violence, which reflects the Eritrean context. Moreover, as the literature highlights, child physical abuse is common in homes where there is aggression towards the spouse [68], and household violence occurs with or leads to household dysfunction in most previous studies [31, 69, 70]. Nonetheless, the infrequency of household dysfunction (e.g., divorce), emotional abuse, and physical neglect in our study could be related to the normalization of aggression particularly towards women and children as a disciplinary act [71]. For example, among the Tigrigna ethnic group in Eritrea (majority of our study’s participants), 8 in 10 wives believe that a husband beating his wife is justified [72], which may reflect the general attitudes towards some actions against women and children. Disciplining the child follows the same norm at home, school, and the community. Hence, there is less household dysfunction despite household violence. Another possible reason could be religious and traditional practices that may protect the functional integrity of the household despite challenges. However, to gain a deeper understanding and tailor culturally sensitive interventions, further investigation of ACEs in the household context is necessary.

Our findings also revealed that CSEs mediated the effects of household violence and multiple ACEs (relative to low ACEs) on psychological distress and subjective well-being. Consistent with prior research, cumulated stressful life events played a mediating role in the relationship between ACEs and college students’ mental health [21]. Moreover, the multiple ACE class exerted a stronger impact on CSEs, compared to the household violence class, which may implicate the well-established cumulative effect of ACEs. Our results also indicated that CSEs exerted more indirect effects on outcomes than ACEs’ direct effects. The literature shows that childhood adversity disrupts systems of stress processes and reduces the threshold for reactivity and coping, or adaptive mechanisms to upcoming stressors. More importantly, in this mechanism, childhood adversity and stress during adulthood can each generate mental health problems or leave neurobiological imprints that exaggerate future reactivity towards stressors [73]. Likewise, the stress proliferation theory posits that ACEs together with CSEs or CSEs in place of ACEs cause harmful health consequences [74]. Consistent with prior studies, the impact was stronger as the risk became more recent [75, 76]. ACEs and CSEs were assumed to be distal and proximal life adversities in the current study, which may explain why CSEs’ indirect effects were greater than ACEs’ direct effects.

Our work furthers the understanding of ACE extent, patterns, and possible conduits through which they exert deleterious impacts on the mental health of Eritrean young adults. However, several limitations should be noted. First, the generalizability of our findings is limited to college students from low-resourced and post-war contexts. Second, the causal relationship between ACEs, CSEs, and outcomes cannot be established, owing to the cross-sectional nature of data. More longitudinal studies are needed to understand ACE classes and their association to mental health. Third, retrospective self-reported ACEs coupled with the conservative culture of our respondents may lead to underreporting of ACEs.

## Conclusions

The current research identified three ACE patterns and discovered that CSEs could mediate the effects of ACE patterns on mental health outcomes. The importance of understanding multiple ACE exposures and their impacts on mental health through CSEs are highlighted by our findings. Therefore, early recognition of ACEs and prevention of further stressors may curb the proliferation of stress and improve mental health.

### Supplementary Information

**Additional file 1:.** Supplementary material

## Data Availability

Data are available from the corresponding author on reasonable request.

## Appendix 1

**Table 6:** Adverse Childhood Experiences International Questionnaire (ACE-IQ)

Category	Questions	Response	
P1	Did your parents/guardians understand your problems and worries?	Always Most of the time Sometimes Rarely Never	1 2 3 4 5
P2	Did your parents/guardians **really** know what you were doing with your free time when you were not at school or work?	Always Most of the time Sometimes Rarely Never	1 2 3 4 5
P3	How often did your parents/guardians **not** give you enough food even when they could easily have done so?	Many times A few times Once Never	1 2 3 4
P4	Were your parents/guardians too drunk or intoxicated () by alcohol/drugs to take care of you?	Many times A few times Once Never	1 2 3 4
P5	How often did your parents/guardians **not** send you to school even when it was available?	Many times A few times Once Never	1 2 3 4
F1	Did you live with a household member who was a problem drinker () or alcoholic?	Yes No	1 2
F2	Did you live with a household member who was depressed, mentally ill, or suicidal?	Yes No	1 2
F3	Did you live with a household member who was ever sent to jail or prison?	Yes No	1 2
F4	Were your parents ever separated or divorced?	Yes No	1 2
F5	Did your mother, father, or guardian die?	Yes No	1 2
F6	Did you see or hear a parent or household member in your home being yelled () at, screamed at and sworn () at, insulted, or humiliated ()?	Many times A few times Once Never	1 2 3 4
F7	Did you see or hear a parent or household member in your home being slapped (), kicked, punched (), or beaten up?	Many times A few times Once Never	1 2 3 4
F8	Did you see or hear a parent or household member in your home being hit or cut with an object, such as a stick (or cane), bottle, knife, belt (), whip () etc.?	Many times A few times Once Never	1 2 3 4
A1	Did a parent, guardian, or other household member yell, scream or swear at you, insult, or humiliate you?	Many times A few times Once Never	1 2 3 4
A2	Did a parent, guardian, or other household member threaten to, or actually, abandon ) you or throw you out of the house?	Many times A few times Once Never	1 2 3 4
A3	Did a parent, guardian, or other household member, slap, kick, punch (), or beat you up?	Many times A few times Once Never	1 2 3 4
A4	Did a parent, guardian or other household member hit or cut you with an object, such as a stick, bottle, knife, belt, whip, etc.?	Many times A few times Once Never	1 2 3 4
A5	Did someone touch or fondle () you in a sexual way when you did not want them to?	Many times A few times Once Never	1 2 3 4
A6	Did someone make you touch their body in a sexual way when you did not want them to?	Many times A few times Once Never	1 2 3 4
A7	Did someone attempt sexual intercourse with you when you did not want them to?	Many times A few times Once Never	1 2 3 4
A8	Did someone actually have sexual intercourse with you when you did not want them to?	Many times A few times Once Never	1 2 3 4
V1	Did other kids, including brothers or sisters, hit you, threaten you, or insult you?	Many times A few times Once Never	1 2 3 4
V2	Did you see or hear someone being beaten up in real life?	Many times A few times Once Never	1 2 3 4
V3	Did you see or hear someone being stabbed () or shot () in real life?	Many times A few times Once Never	1 2 3 4
V4	Did you see or hear someone being threatened () with a knife or gun in real life?	Many times A few times Once Never	1 2 3 4
V5	Were you forced to go and live in another place due to any of these events?	Many times A few times Once Never	1 2 3 4
V6	Did you experience the deliberate () destruction of your home due to any of these events?	Many times A few times Once Never	1 2 3 4
V7	Were you beaten up by soldiers, police, militia, or gangs?	Many times A few times Once Never	1 2 3 4
V8	Was a family member or friend killed or beaten up by soldiers, police, militia, or gangs?	Many times A few times Once Never	1 2 3 4

## Appendix 2

**Table 7:** Tetrachoric correlations between items of ACE-IQ

Items	1	2	3	4	5	6	7	8	9	10	11	12
1-Physical abuse	-											
2-Emotional abuse	0.64	-										
3-Sexual abuse	0.17	0.29	-									
4-Household substance abuse	0.19	0.33	0.27	-								
5-Incarcerated family member	0.35	0.29	0.27	0.59	-							
6-Household mental illness	0.13	0.36	0.27	0.36	0.16	-						
7-Domestic violence	0.56	0.59	0.30	0.31	0.37	0.15	-					
8-Parental separation/divorce	0.10	0.21	0.13	0.02	0.17	0.18	0.19	-				
9-Emotional neglect	0.10	0.38	0.16	0.20	0.24	−0.01	0.25	0.28	-			
10-Physical neglect	0.17	0.21	0.24	0.47	0.22	0.13	0.24	0.09	0.13	-		
11-Bullying	0.60	0.59	0.31	0.18	0.32	0.28	0.30	0.18	0.33	0.23	-	
12-Community violence	0.35	0.29	0.19	0.17	0.22	0.17	0.25	0.15	0.16	0.25	0.29	-
13-Collective violence	0.20	0.12	0.20	0.05	0.17	0.28	0.23	0.15	0.17	0.14	0.17	0.25

## Appendix 3

**Table 8:** Current stressful events

No.	Questions	Response	
1	Death of a close family member	Yes No	1 0
2	Serious illness/injury to close family member	Yes No	1 0
3	Serious illness/injury to you	Yes No	1 0
4	Divorce of parents	Yes No	1 0
5	Family member arrested	Yes No	1 0
6	Serious break-up with boyfriend/girlfriend	Yes No	1 0
7	Family has major financial pressures	Yes No	1 0
8	You having major financial pressures	Yes No	1 0
9	Addiction ()/psychological struggle of family member	Yes No	1 0
10	You struggling with addiction/psychological problem	Yes No	1 0
11	Cheated on by boyfriend/girlfriend	Yes No	1 0
12	Parents have ongoing conflicts	Yes No	1 0
13	You having ongoing conflict with parents	Yes No	1 0
14	You experiencing abuse/violence at home	Yes No	1 0
15	Unwanted sexual behavior imposed on you	Yes No	1 0
16	Unwanted pregnancy (either you or you being the father)	Yes No	1 0
17	Increased workload at college	Yes No	1 0
18	Having to repeat a course	Yes No	1 0
19	Lower grades than expected	Yes No	1 0
20	I am away from home and feel lonely	Yes No	1 0

## Appendix 4

**Table 9:** Tetrachoric correlations between items of CSEs scale

Items	1	2	3	4	5	6	7	8	9	10	11	12	13	14	15	16	17	18
1-Household death	-																	
2-Household illness	0.34	-																
3-Individual illness	0.15	0.44	-															
4-Parental divorce	0.26	0.26	0.47	-														
5-Arrested family member	0.27	0.32	0.24	0.42	-													
6-Break-up with boyfriend/girlfriend	0.17	0.10	0.11	0.19	0.24	-												
7-Household financial pressures	0.09	0.19	0.12	0.23	0.08	0.06	-											
8-Individual financial pressures	0.07	0.18	0.06	0.24	0.09	0.05	0.82	-										
9-Household mental illness	−0.03	0.11	0.11	−0.03	0.21	0.19	0.33	0.30	-									
10-Individual mental illness	−0.07	0.14	0.29	0.13	0.15	0.21	0.22	0.37	0.50	-								
11-Cheated by boyfriend/girlfriend	0.09	0.24	0.19	0.28	0.21	0.61	−0.12	0.07	0.40	0.51	-							
12-Parental conflicts	−0.07	0.18	0.31	0.55	0.33	0.06	0.24	0.31	0.38	0.39	0.22	-						
13-Conflicts with parents	0.17	0.17	0.25	0.14	0.33	0.25	0.25	0.36	0.28	0.44	0.24	0.65	-					
14-Abuse/violence	−0.02	−0.09	0.21	0.35	0.11	0.24	0.19	0.28	0.35	0.36	0.47	0.31	0.53	-				
15-Unwanted sexual behavior	0.04	0.08	0.26	0.09	0.02	0.09	0.14	0.22	0.15	0.05	0.17	0.10	0.07	0.05	-			
16-Unwanted pregnancy	0.07	0.21	0.15	0.11	0.06	0.27	0.16	0.17	0.05	0.23	0.08	0.20	0.07	0.17	0.14	-		
17-Increased workload	0.07	0.03	0.29	0.19	0.20	0.26	−0.03	0.16	0.26	0.12	0.23	0.06	0.19	0.25	0.32	0.36	-	
18-Course failure	−0.03	0.21	0.07	−0.05	0.05	0.11	0.10	0.20	0.07	0.16	−0.08	0.01	0.09	0.09	0.18	0.26	0.01	-
20-Feel lonely	0.04	0.26	−0.08	0.10	0.11	0.26	0.17	0.22	0.26	0.16	0.34	0.06	0.19	0.31	0.06	0.05	−0.06	0.11

## Appendix 5

**Table 10:** Tetrachoric correlations between items of SRQ-20

Items	1	2	3	4	5	6	7	8	9	10	11	12	13	14	15	16	17	18	19
1-Headaches	-																		
2-Appetite	0.26	-																	
3-Sleep	0.27	0.31	-																
4-Frightened	0.25	0.24	0.49	-															
5-Hands shake	0.34	0.17	0.34	0.37	-														
6-Nervous	0.45	0.21	0.50	0.64	0.52	-													
7-Digestion	0.34	0.50	0.36	0.23	0.29	0.17	-												
8-Thinking problems	0.34	0.11	0.44	0.36	0.34	0.44	0.28	-											
9-Unhappy	0.29	0.13	0.39	0.40	0.29	0.59	0.17	0.54	-										
10-Crying	0.37	0.11	0.21	0.46	0.36	0.40	0.16	0.38	0.33	-									
11-Daily activities	0.30	0.19	0.34	0.35	0.26	0.37	0.30	0.40	0.56	0.26	-								
12-Making decisions	0.36	0.18	0.24	0.36	0.21	0.47	0.22	0.53	0.48	0.46	0.49	-							
13-Daily work	0.06	0.14	0.28	0.18	0.26	0.16	0.31	0.28	0.27	0.20	0.47	0.23	-						
14-Useless	0.29	0.11	0.38	0.40	0.32	0.36	0.20	0.39	0.52	0.30	0.52	0.56	0.39	-					
15-Lost interest	0.34	0.17	0.26	0.38	0.32	0.42	0.27	0.32	0.43	0.32	0.49	0.45	0.17	0.47	-				
16-Worthless	0.30	0.18	0.33	0.40	0.32	0.45	0.10	0.44	0.54	0.44	0.38	0.45	0.29	0.50	0.47	-			
17-Suicide idea	0.19	0.19	0.29	0.28	0.19	0.26	0.31	0.28	0.32	0.29	0.26	0.21	0.21	0.34	0.14	0.41	-		
18-Always tired	0.26	0.24	0.36	0.28	0.09	0.21	0.31	0.29	0.20	0.24	0.38	0.24	0.32	0.22	0.36	0.29	0.33	-	
19-Easily tired	0.28	0.21	0.28	0.28	0.29	0.21	0.44	0.32	0.25	0.29	0.25	0.34	0.35	0.32	0.33	0.19	0.15	0.56	-
20-Stomach	0.35	0.33	0.29	0.17	0.29	0.27	0.63	0.23	0.31	0.22	0.31	0.32	0.22	0.26	0.30	0.24	0.24	0.21	0.38

## Abbreviations

ACEs: Adverse Childhood Experiences
CSEs: Current Stressful Events
LCA: Latent Class Analysis
ACE-IQ: Adverse Childhood Experience International Questionnaire
CD-RISC-10: 10-item Connor Davidson Resilience Scale
SRQ-20: 20-item Self-Reporting Questionnaire
WHO-5: 5-item World Health Organization Well-Being Index
AIC: Akaike’s Information Criteria
BIC: Bayesian Information Criteria
aBIC: Adjusted Bayesian Information Criteria
BLRT: Bootstrap Likelihood Ratio Test
CI: Confidence Interval
SD: Standard Deviation
SE: Standard Error
