# Correction to: Classes of childhood adversities and their associations to the mental health of college undergraduates: a nationwide cross-sectional study

**DOI:** 10.1186/s12199-021-01003-6

**Published:** 2021-08-19

**Authors:** Peigang Wang, Mohammedhamid Osman Kelifa, Bin Yu, Yinmei Yang

**Affiliations:** 1 School of Health Sciences, Wuhan University, 115 Donghu Road, Wuhan City, 430,071 Hubei Province China; 2 Orotta College of Medicine and Health Sciences, Asmara, Meakel Eritrea; 3 Department of Surgery, Duke University, Durham, NC 27710 USA

**Correction to: Environ Health Prev Med 26, 73 (2021)**

**https://doi.org/10.1186/s12199-021-00993-7**

Following the publication of the original article [1] the authors noticed that the published Fig. 2 is wrong. The original article [1] has been updated.

Below is the correct Fig. 2.

**Fig. 2:**
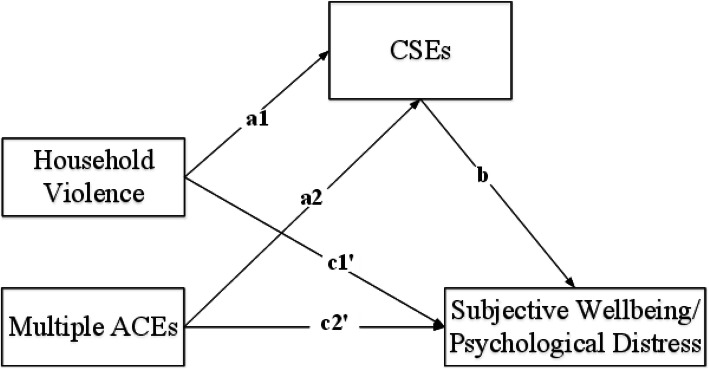
Multicategorical mediation model for psychological distress/subjective well-being. a1 and a2 represent the effects of household violence and multiple ACEs on CSEs, compared to low ACEs; b represents the effect of CSEs on psychological distress/subjective well-being; c1' and c2' represent the relative direct effects of household violence and multiple ACEs on psychological distress/subjective well-being, relative to low ACEs. *CSEs* current stressful events, *ACEs* adverse childhood experiences. All paths are unstandardized coefficients
