# Supplementary material for: Classes of childhood adversities and their associations to the mental health of college undergraduates: a nationwide cross-sectional study
Source: Environ Health Prev Med. 2021 Jul 17;26:73. doi: 10.1186/s12199-021-00993-7 (PMC8286578; doi:10.1186/s12199-021-00993-7)
Supplement: Supplementary file 1 — Additional file 1:. Supplementary material [file 12199_2021_993_MOESM1_ESM.docx]

**Table 1** Proportion by year

| Year | Population | Proportion | Sample Size |
| --- | --- | --- | --- |
| 1^st^ | 1807 | 0.315(1807/5740) | 178 |
| 2^nd^ | 1536 | 0.267(1536/5740) | 151 |
| 3^rd^ | 1415 | 0.246(1415/5740) | 139 |
| 4^th^ | 838 | 0.145(838/5740) | 82 |
| 5^th^ | 121 | 0.021(121/5740) | 12 |
| 6^th^ | 23 | 0.004(23/5740) | 2 |
| Population | 5740 | - | 564 |
| Sample Size | 564 | - | - |

Note: 5^th^ year are engineering medicine, 6^th^ year only medicine

**Table 2** Adverse Childhood Experiences International Questionnaire (ACE-IQ)

| Category | Questions | Response |
| --- | --- | --- |
| P1 | Did your parents/guardians understand your problems and worries? | Always 1  Most of the time 2  Sometimes 3  Rarely 4  Never 5 |
| P2 | Did your parents/guardians **really** know what you were doing with your free time when you were not at school or work? | Always 1  Most of the time 2  Sometimes 3  Rarely 4  Never 5 |
| P3 | How often did your parents/guardians **not** give you enough food even when they could easily have done so? | Many times 1  A few times 2  Once 3  Never 4 |
| P4 | Were your parents/guardians too drunk or intoxicated (ሰኺሩ) by alcohol/drugs to take care of you? | Many times 1  A few times 2  Once 3  Never 4 |
| P5 | How often did your parents/guardians **not** send you to school even when it was available? | Many times 1  A few times 2  Once 3  Never 4 |
| F1 | Did you live with a household member who was a problem drinker (ሰታይ) or alcoholic? | Yes 1  No 2 |
| F2 | Did you live with a household member who was depressed, mentally ill or suicidal? | Yes 1  No 2 |
| F3 | Did you live with a household member who was ever sent to jail or prison? | Yes 1  No 2 |
| F4 | Were your parents ever separated or divorced? | Yes 1  No 2 |
| F5 | Did your mother, father or guardian die? | Yes 1  No 2 |
| F6 | Did you see or hear a parent or household member in your home being yelled (ጨደረ) at, screamed at and sworn (ተጻሪፉ) at, insulted or humiliated (ኣሕፈረ)? | Many times 1  A few times 2  Once 3  Never 4 |
| F7 | Did you see or hear a parent or household member in your home being slapped (ተጸፈዐ), kicked, punched (ኣጉሰጠ) or beaten up? | Many times 1  A few times 2  Once 3  Never 4 |
| F8 | Did you see or hear a parent or household member in your home being hit or cut with an object, such as a stick (or cane), bottle, knife, belt (ቁልፊ)፡ whip (ኩርማጅ) etc.? | Many times 1  A few times 2  Once 3  Never 4 |
| A1 | Did a parent, guardian or other household member yell, scream or swear at you, insult or humiliate you? | Many times 1  A few times 2  Once 3  Never 4 |
| A2 | Did a parent, guardian or other household member threaten to, or actually, abandon (ጠንጢኑ፡ ገዲፉ) you or throw you out of the house? | Many times 1  A few times 2  Once 3  Never 4 |
| A3 | Did a parent, guardian or other household member, slap, kick, punch (ኣጉሰጠ) or beat you up? | Many times 1  A few times 2  Once 3  Never 4 |
| A4 | Did a parent, guardian or other household member hit or cut you with an object, such as a stick, bottle, knife, belt, whip etc? | Many times 1  A few times 2  Once 3  Never 4 |
| A5 | Did someone touch or fondle (ደረዘ) you in a sexual way when you did not want them to? | Many times 1  A few times 2  Once 3  Never 4 |
| A6 | Did someone make you touch their body in a sexual way when you did not want them to? | Many times 1  A few times 2  Once 3  Never 4 |
| A7 | Did someone attempt sexual intercourse with you when you did not want them to? | Many times 1  A few times 2  Once 3  Never 4 |
| A8 | Did someone actually have sexual intercourse with you when you did not want them to? | Many times 1  A few times 2  Once 3  Never 4 |
| V1 | Did other kids, including brothers or sisters, hit you, threaten you or insult you? | Many times 1  A few times 2  Once 3  Never………………………….4 |
| V2 | Did you see or hear someone being beaten up in real life? | Many times 1  A few times 2  Once 3  Never 4 |
| V3 | Did you see or hear someone being stabbed (ብካራ ወይ ካልእ በሊሕ ነገር ክውጋእ እንከሎ) or shot (ብጥይት ክህረም እንከሎ) in real life? | Many times 1  A few times 2  Once 3  Never 4 |
| V4 | Did you see or hear someone being threatened (ምፍርራሕ) with a knife or gun in real life? | Many times 1  A few times 2  Once 3  Never 4 |
| V5 | Were you forced to go and live in another place due to any of these events? | Many times 1  A few times 2  Once 3  Never 4 |
| V6 | Did you experience the deliberate (ብደይመደይ) destruction of your home due to any of these events? | Many times 1  A few times 2  Once 3  Never 4 |
| V7 | Were you beaten up by soldiers, police, militia, or gangs? | Many times 1  A few times 2  Once 3  Never 4 |
| V8 | Was a family member or friend killed or beaten up by soldiers, police, militia, or gangs? | Many times 1  A few times 2  Once 3  Never 4 |

**Table 3**  Descriptive statistics of SRQ-20 scale

| Item | Mean | Variance | Skewness | Kurtosis | Discrimination | Difficulty |
| --- | --- | --- | --- | --- | --- | --- |
| 1-headaches | 0.42 | 0.24 | 0.32 | -1.91 | 1.02 | 0.38 |
| 2-appetite | 0.37 | 0.23 | 0.54 | -1.72 | 0.61 | 0.94 |
| 3-sleep | 0.32 | 0.22 | 0.76 | -1.43 | 1.27 | 0.77 |
| 4-frightened | 0.25 | 0.19 | 1.19 | -0.58 | 1.46 | 1.07 |
| 5-hands shake | 0.13 | 0.11 | 2.23 | 2.99 | 1.19 | 1.99 |
| 6-nervous | 0.48 | 0.25 | 0.09 | -2.00 | 1.67 | 0.10 |
| 7-digestion | 0.17 | 0.14 | 1.77 | 1.12 | 1.01 | 1.87 |
| 8-thinking problems | 0.31 | 0.21 | 0.83 | -1.32 | 1.49 | 0.76 |
| 9-unhappy | 0.38 | 0.24 | 0.49 | -1.76 | 1.75 | 0.44 |
| 10-crying | 0.24 | 0.18 | 1.24 | -0.46 | 1.17 | 1.26 |
| 11-daily activities | 0.37 | 0.23 | 0.55 | -1.71 | 1.55 | 0.51 |
| 12-making decisions | 0.46 | 0.25 | 0.16 | -1.98 | 1.48 | 0.17 |
| 13-daily work | 0.24 | 0.18 | 1.23 | -0.49 | 0.88 | 1.53 |
| 14-useless | 0.27 | 0.20 | 1.04 | -0.93 | 1.60 | 0.90 |
| 15-lost interest | 0.52 | 0.25 | -0.07 | -2.00 | 1.33 | -0.06 |
| 16-worthless | 0.21 | 0.17 | 1.42 | 0.02 | 1.58 | 1.18 |
| 17-suicide idea | 0.07 | 0.07 | 3.29 | 8.88 | 1.07 | 2.78 |
| 18-always tired | 0.30 | 0.21 | 0.87 | -1.26 | 0.93 | 1.07 |
| 19-easily tired | 0.35 | 0.23 | 0.63 | -1.62 | 1.00 | 0.75 |
| 20-stomach | 0.34 | 0.23 | 0.68 | -1.54 | 0.98 | 0.82 |

**Table 4** Descriptive statistics of ACEs scale

| ACEs category | Mean | Variance | Skewness | Kurtosis | Discrimination | Difficulty |
| --- | --- | --- | --- | --- | --- | --- |
| 1-Physical abuse | 0.37 | 0.05 | 3.91 | 13.31 | 2.54 | 1.95 |
| 2-Emotional abuse | 0.09 | 0.24 | -0.33 | -1.90 | 1.57 | -0.30 |
| 3-Sexual abuse | 0.27 | 0.20 | 1.04 | -0.93 | 0.87 | 1.31 |
| 4-Household substance abuse | 0.05 | 0.05 | 4.18 | 15.50 | 1.09 | 3.18 |
| 5-Incarcerated family member | 0.12 | 0.11 | 2.37 | 3.63 | 1.03 | 2.31 |
| 6-Household mental illness | 0.06 | 0.06 | 3.60 | 11.03 | 0.75 | 3.90 |
| 7-Domestic violence | 0.39 | 0.24 | 0.47 | -1.79 | 1.42 | 0.45 |
| 8-Parental separation/divorce | 0.23 | 0.18 | 1.31 | -0.29 | 0.46 | 2.80 |
| 9-Emotional neglect | 0.19 | 0.25 | 0.17 | -1.98 | 0.77 | 0.25 |
| 10-Physical neglect | 0.18 | 0.05 | 3.91 | 13.31 | 0.73 | 4.18 |
| 11-Bullying | 0.06 | 0.06 | 3.60 | 11.03 | 1.68 | 2.23 |
| 12-Community violence | 0.31 | 0.21 | 0.83 | -1.32 | 0.94 | 1.00 |
| 13-Collective violence | 0.40 | 0.24 | 0.40 | -1.85 | 0.59 | 0.72 |

**Table 5** Tetrachoric correlations between items of ACE-IQ.

| Items | 1 | 2 | 3 | 4 | 5 | 6 | 7 | 8 | 9 | 10 | 11 | 12 |
| --- | --- | --- | --- | --- | --- | --- | --- | --- | --- | --- | --- | --- |
| 1-Physical abuse | - |  |  |  |  |  |  |  |  |  |  |  |
| 2-Emotional abuse | 0.64 | - |  |  |  |  |  |  |  |  |  |  |
| 3-Sexual abuse | 0.17 | 0.29 | - |  |  |  |  |  |  |  |  |  |
| 4-Household substance abuse | 0.19 | 0.33 | 0.27 | - |  |  |  |  |  |  |  |  |
| 5-Incarcerated family member | 0.35 | 0.29 | 0.27 | 0.59 | - |  |  |  |  |  |  |  |
| 6-Household mental illness | 0.13 | 0.36 | 0.27 | 0.36 | 0.16 | - |  |  |  |  |  |  |
| 7-Domestic violence | 0.56 | 0.59 | 0.30 | 0.31 | 0.37 | 0.15 | - |  |  |  |  |  |
| 8-Parental separation/divorce | 0.10 | 0.21 | 0.13 | 0.02 | 0.17 | 0.18 | 0.19 | - |  |  |  |  |
| 9-Emotional neglect | 0.10 | 0.38 | 0.16 | 0.20 | 0.24 | -0.01 | 0.25 | 0.28 | - |  |  |  |
| 10-Physical neglect | 0.17 | 0.21 | 0.24 | 0.47 | 0.22 | 0.13 | 0.24 | 0.09 | 0.13 | - |  |  |
| 11-Bullying | 0.60 | 0.59 | 0.31 | 0.18 | 0.32 | 0.28 | 0.30 | 0.18 | 0.33 | 0.23 | - |  |
| 12-Community violence | 0.35 | 0.29 | 0.19 | 0.17 | 0.22 | 0.17 | 0.25 | 0.15 | 0.16 | 0.25 | 0.29 | - |
| 13-Collective violence | 0.20 | 0.12 | 0.20 | 0.05 | 0.17 | 0.28 | 0.23 | 0.15 | 0.17 | 0.14 | 0.17 | 0.25 |

**Table 6** Tetrachoric correlations between items of SRQ-20.

| Items | 1 | 2 | 3 | 4 | 5 | 6 | 7 | 8 | 9 | 10 | 11 | 12 | 13 | 14 | 15 | 16 | 17 | 18 | 19 |
| --- | --- | --- | --- | --- | --- | --- | --- | --- | --- | --- | --- | --- | --- | --- | --- | --- | --- | --- | --- |
| 1-headaches | - |  |  |  |  |  |  |  |  |  |  |  |  |  |  |  |  |  |  |
| 2-appetite | 0.26 | - |  |  |  |  |  |  |  |  |  |  |  |  |  |  |  |  |  |
| 3-sleep | 0.27 | 0.31 | - |  |  |  |  |  |  |  |  |  |  |  |  |  |  |  |  |
| 4-frightened | 0.25 | 0.24 | 0.49 | - |  |  |  |  |  |  |  |  |  |  |  |  |  |  |  |
| 5-hands shake | 0.34 | 0.17 | 0.34 | 0.37 | - |  |  |  |  |  |  |  |  |  |  |  |  |  |  |
| 6-nervous | 0.45 | 0.21 | 0.50 | 0.64 | 0.52 | - |  |  |  |  |  |  |  |  |  |  |  |  |  |
| 7-digestion | 0.34 | 0.50 | 0.36 | 0.23 | 0.29 | 0.17 | - |  |  |  |  |  |  |  |  |  |  |  |  |
| 8-thinking problems | 0.34 | 0.11 | 0.44 | 0.36 | 0.34 | 0.44 | 0.28 | - |  |  |  |  |  |  |  |  |  |  |  |
| 9-unhappy | 0.29 | 0.13 | 0.39 | 0.40 | 0.29 | 0.59 | 0.17 | 0.54 | - |  |  |  |  |  |  |  |  |  |  |
| 10-crying | 0.37 | 0.11 | 0.21 | 0.46 | 0.36 | 0.40 | 0.16 | 0.38 | 0.33 | - |  |  |  |  |  |  |  |  |  |
| 11-daily activities | 0.30 | 0.19 | 0.34 | 0.35 | 0.26 | 0.37 | 0.30 | 0.40 | 0.56 | 0.26 | - |  |  |  |  |  |  |  |  |
| 12-making decisions | 0.36 | 0.18 | 0.24 | 0.36 | 0.21 | 0.47 | 0.22 | 0.53 | 0.48 | 0.46 | 0.49 | - |  |  |  |  |  |  |  |
| 13-daily work | 0.06 | 0.14 | 0.28 | 0.18 | 0.26 | 0.16 | 0.31 | 0.28 | 0.27 | 0.20 | 0.47 | 0.23 | - |  |  |  |  |  |  |
| 14-useless | 0.29 | 0.11 | 0.38 | 0.40 | 0.32 | 0.36 | 0.20 | 0.39 | 0.52 | 0.30 | 0.52 | 0.56 | 0.39 | - |  |  |  |  |  |
| 15-lost interest | 0.34 | 0.17 | 0.26 | 0.38 | 0.32 | 0.42 | 0.27 | 0.32 | 0.43 | 0.32 | 0.49 | 0.45 | 0.17 | 0.47 | - |  |  |  |  |
| 16-worthless | 0.30 | 0.18 | 0.33 | 0.40 | 0.32 | 0.45 | 0.10 | 0.44 | 0.54 | 0.44 | 0.38 | 0.45 | 0.29 | 0.50 | 0.47 | - |  |  |  |
| 17-suicide idea | 0.19 | 0.19 | 0.29 | 0.28 | 0.19 | 0.26 | 0.31 | 0.28 | 0.32 | 0.29 | 0.26 | 0.21 | 0.21 | 0.34 | 0.14 | 0.41 | - |  |  |
| 18-always tired | 0.26 | 0.24 | 0.36 | 0.28 | 0.09 | 0.21 | 0.31 | 0.29 | 0.20 | 0.24 | 0.38 | 0.24 | 0.32 | 0.22 | 0.36 | 0.29 | 0.33 | - |  |
| 19-easily tired | 0.28 | 0.21 | 0.28 | 0.28 | 0.29 | 0.21 | 0.44 | 0.32 | 0.25 | 0.29 | 0.25 | 0.34 | 0.35 | 0.32 | 0.33 | 0.19 | 0.15 | 0.56 | - |
| 20-stomach | 0.35 | 0.33 | 0.29 | 0.17 | 0.29 | 0.27 | 0.63 | 0.23 | 0.31 | 0.22 | 0.31 | 0.32 | 0.22 | 0.26 | 0.30 | 0.24 | 0.24 | 0.21 | 0.38 |

**Table 7** Associations between ACEs classes and mental health [Mean (SD)]

|  | ACEs classes | | | F | p |
| --- | --- | --- | --- | --- | --- |
|  | Low  ACEs | Household Violence | Multiple ACEs |  |  |
| Psychological distress | 5.83 (4.28) | 6.03 (4.00) | 8.04 (4.26) | 8.343 | <0.001 |
| Subjective wellbeing | 15.05 (5.02) | 14.93 (5.35) | 13.46 (5.39) | 3.021 | 0.045 |
